# A real-world case–control study on the efficacy and safety of pulsed field ablation for atrial fibrillation

**DOI:** 10.1186/s40001-023-01509-5

**Published:** 2023-11-15

**Authors:** Ming Yang, Peng-yu Wang, Ying-lu Hao, Mei Liang, Zi-yang Yu, Xi-chen Li, Yan-ping Li

**Affiliations:** Department of Cardiology, The People’s Hospital of Yuxi City, The 6th Affiliated Hospital of Kunming Medical University, No.21 of Nieer Road, Hongta District, Yuxi, 653100 China

**Keywords:** Atrial fibrillation, Pulsed field ablation, Radiofrequency ablation

## Abstract

**Objective:**

The primary objective of this study was to evaluate the efficacy and safety of pulsed field ablation in individuals diagnosed with atrial fibrillation.

**Methods:**

A total of 36 patients diagnosed with atrial fibrillation were enrolled in the pulsed field ablation group, while another 36 patients diagnosed with atrial fibrillation were included in the radiofrequency ablation group. Among the study participants, 15 patients in the pulsed field ablation group and 17 patients in the radiofrequency ablation group had persistent atrial fibrillation. Comprehensive comparisons were made between the two groups, including baseline data, underlying diseases, medication usage, intraoperative parameters, and atrial fibrillation recurrence rates at 1, 3, and 6 months during the postoperative follow-up period.

**Results:**

(1) There were no significant differences observed between the two groups concerning baseline data and antiarrhythmic drug usage (*P* > 0.05); (2) the effective ablation time for both left and right pulmonary veins in the pulsed field ablation group was markedly shorter compared to the radiofrequency ablation group (*P* < 0.001 for each vein); (3) within the pulsed field ablation group, the number of discharges, catheter operation time, and effective ablation time for the left pulmonary vein were significantly higher than those for the right pulmonary vein (*P* < 0.05). Conversely, in the radiofrequency ablation group, the number of discharges for the left pulmonary vein was significantly higher than that for the right pulmonary vein (*P* < 0.05); and (4) when comparing sinus rhythm maintenance at 1, 3, and 6 months postoperatively, no statistically significant differences were noted between the two groups for paroxysmal, persistent, and paroxysmal + persistent atrial fibrillation cases (*P* > 0.05).

**Conclusion:**

During the 6-month follow-up period, pulsed field ablation demonstrated comparable efficacy to radiofrequency ablation with respect to recurrence rates for both paroxysmal and persistent atrial fibrillation. Moreover, pulsed field ablation exhibited high safety levels, excellent surgical efficiency, and a notably brief learning curve, affirming its viability as a therapeutic option for these conditions.

## Introduction

Atrial fibrillation (AF) stands as one of the most prevalent clinical arrhythmias, with its incidence steadily increasing in parallel with the aging of the global population [1, 2]. This rise has led to substantial economic and health burdens. AF ablation has emerged as a viable and effective therapeutic approach for managing AF, a recommendation endorsed both by Chinese medical guidelines and international standards [3, 4]. Nevertheless, prevailing methods like radiofrequency ablation and cryoablation lead to coagulative necrosis of tissues that come into contact with the heat from radiofrequency energy or the low temperatures from liquid nitrogen cryoplasty. These techniques lack tissue specificity, potentially causing harm to neighboring structures like the phrenic nerve, esophagus, or coronary arteries. Pulsed field ablation (PFA) represents an innovative ablation technique, involving the induction of electroporation by generating pores in the phospholipid bilayer of cell membranes through intermittent high-intensity electric fields, which occur over a brief duration (microseconds) [5, 6]. Electroporation exerts a graded effect, ranging from temporary reversible alterations to irreversible outcomes such as apoptosis and necrosis [7]. The cellular impact observed is contingent upon factors such as pulse duration, voltage, frequency, and polarity of the applied electric field. Hence, pulsed low-intensity electric fields lead to the formation of reversible and transient pores (reversible electroporation), enabling drug or gene delivery without compromising cell viability. Conversely, electric fields of higher intensity induce substantial alterations in membrane permeability, leading to permanent damage and eventual apoptosis [8, 9]. Previous clinical studies involving animal experiments have shown that PFA displays tissue selectivity, thus reducing the potential for causing lasting damage to neighboring tissues proximate to the atrial muscle. This technique has also displayed a notably high success rate in achieving both immediate and long-term pulmonary vein isolation, effectively averting potential stenosis [10, 11]. The efficacy and safety of PFA for ablation in patients with paroxysmal and persistent AF, however, have not yet been elucidated.

## Materials and methods

### Study participants

Between November 2021 and November 2022, a total of 36 patients, encompassing 128 pulmonary veins, were included in the PFA group. These patients underwent PFA procedures guided by the three-dimensional magnetic navigation system (LEAD-Mapping system, provided by Sichuan Jinjiang Electronic Technology Co., Ltd.) at the Department of Cardiology, Sixth Affiliated Hospital of Kunming Medical University. Propensity score matching was conducted using the baseline values of the PFA group. Subsequently, 36 patients undergoing radiofrequency ablation for AF guided by the three-dimensional electro-anatomical mapping system (Carto3, Biosense-Webster) were identified and enrolled in the radiofrequency ablation group. All patients provided their informed consent and the study was approved by the ethics committee.

### Inclusion criteria

- 1. Patients with paroxysmal AF (defined as AF terminating spontaneously or with intervention within 7 days of onset) and persistent AF (defined as continuously sustained AF beyond 7 days, including episodes terminated by cardioversion, either through medications or electrical means, after ≥ 7 days), aged 18 years or older, who are unable to tolerate class I–IV antiarrhythmic drugs or do not respond to medical therapy.
- 2. Participants with a left atrial size not exceeding 50 mm.

### Exclusion criteria

- 1. Patients with a history of prior radiofrequency ablation.
- 2. Patients diagnosed with severe cardiac insufficiency (left ventricular ejection fraction, LVEF ≤ 35%).
- 3. Patients suffering from severe renal insufficiency (estimated glomerular filtration rate, eGFR < 30 ml/min).
- 4. Patients with immune system disorders or blood-related illnesses.
- 5. Patients currently experiencing acute infections or diagnosed with chronic inflammatory diseases.
- 6. Patients diagnosed with malignant tumors.
- 7. Individuals unwilling or unable to provide informed consent.
- 8. Patients with AF secondary to an electrolyte imbalance, thyroid disease, or reversible or non-cardiac causes.
- 9. Patients diagnosed with atrial thrombosis.

### Electrophysiological examination and PFA

Local anesthesia was administered for non-ultrasound-guided femoral vein puncture. A 10-pole electrode was carefully positioned through the femoral vein to the coronary sinus. Atrial septal puncture was routinely conducted through the right femoral vein using the atrial septal puncture sheath. Subsequently, a loading dose of heparin (100 U/kg) was injected through the sheath, followed by intermittent heparin administration as needed. During the procedure, the activation coagulation duration was maintained within the range of 300 to 350 s. An 8F disposable cardiac PFA catheter (PFA8D18L, Sichuan Jinjiang Electronic Technology Co., Ltd., with a diameter of 15 mm and 7 electrodes) was carefully inserted through the sheath into the left atrium. Using the three-dimensional magnetic navigation system (LEAD-Mapping system, Sichuan Jinjiang Electronic Technology Co., Ltd.), a left atrial model and voltage mapping were meticulously created. Bilateral pulmonary venography was conducted through the sheath, and the pulsed catheter was guided to the pulmonary vein for the ablation procedure. Pacing was performed with the S1S1 protocol (with a shorter than normal RR interval, ranging from 400 to 600 ms and incrementing in 5 mA steps from 5 to 15 mA) utilizing the coronary sinus electrodes and the pulsed catheter electrode pacing (both set at the highest potential). This pacing protocol was employed to assess bidirectional conduction and blockage between the left atrium and pulmonary veins.

**Fig. 1:**
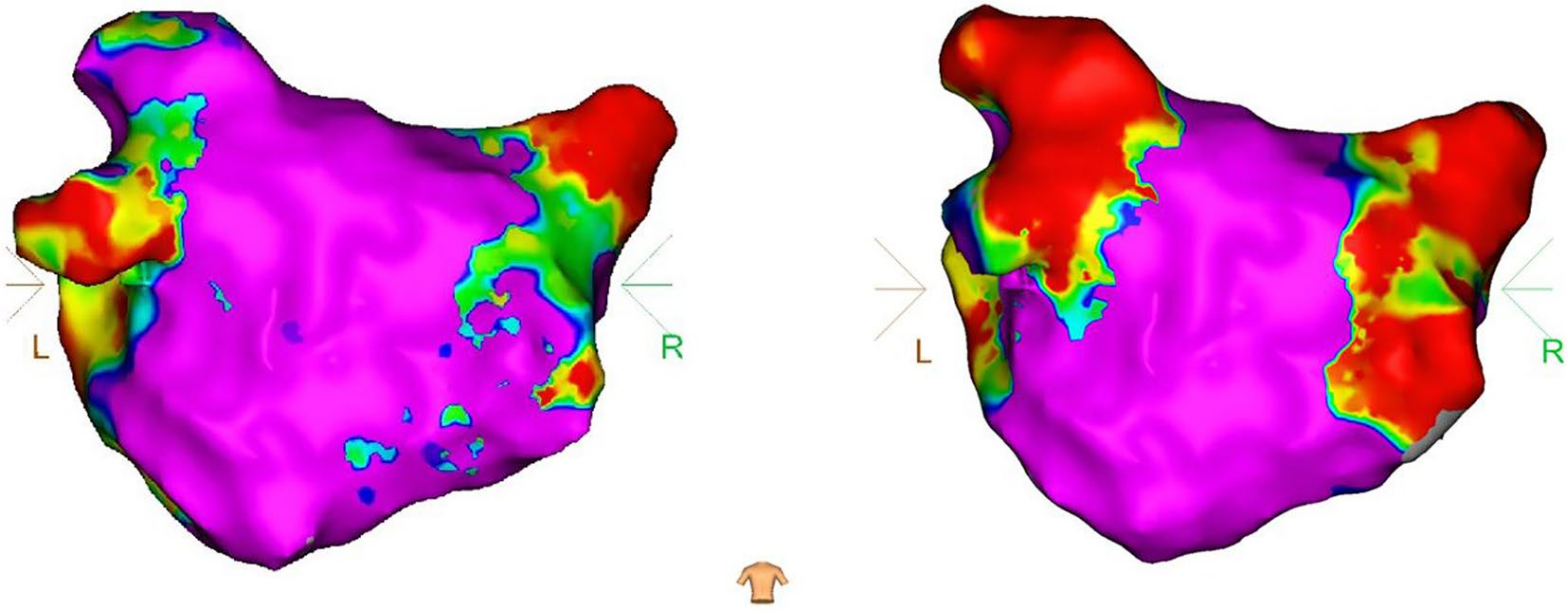
Comparison of PA position matrix mapping before and after ablation (left: before ablation, right: after ablation)

**Fig. 2:**
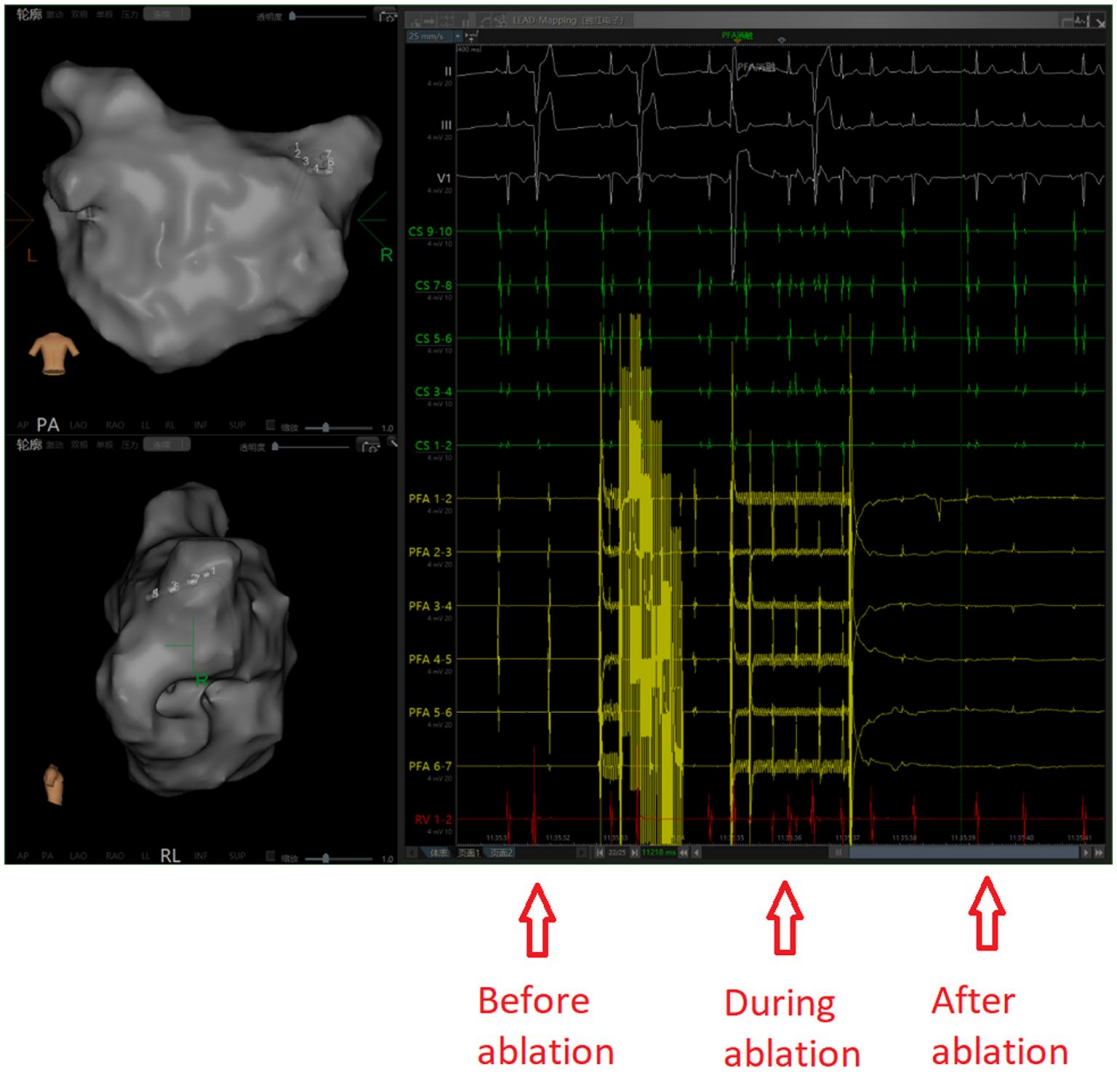
Pre- and post-ablation potentials (left: pre-ablation potential, right: post-ablation potential)

A similar approach was adopted for posterior left atrial wall pacing to assess bidirectional block. Subsequently, investigations were carried out to explore the feasibility of linear ablation. The selection of the ablation line was determined by the surgeon’s proficiency and the degree of atrial fibrosis. Intraoperative sedation was maintained through routine administration of fentanyl. The left and right pulmonary veins were isolated sequentially using the cardiac PFA instrument (LEAD-PFA, Sichuan Jinjiang Electronic Technology Co., Ltd.) and annular pulsed catheter. A voltage amplitude of 1800 V with a discharge time of 4.48 s was applied during the PFA procedure. Five sets of pulses were initiated with an interval of 400 ms. Any voltage measurement less than 0.5 mV before and after ablation was considered indicative of poor adhesion or a low voltage area. The effective pulse ablation time was 480 ms, as illustrated in Figs. 1 and 2.

Following pulmonary vein isolation (PVI), the previously mentioned methods were repeated to perform pacing in the pulmonary vein, left atrial posterior wall, and the coronary sinus. Bidirectional conduction and block between the left atrium and the pulmonary vein, as well as the isolation of the left atrial posterior wall were re-evaluated. Voltage mapping was utilized to assess the state of isolation. For non-isolated pulmonary veins, ablation was reinforced, and linear ablation continued until successful isolation or blockage was confirmed through testing.

### Observation indicators

Efficacy endpoints included various parameters such as operation time, duration of catheter retention in the left atrium, radiation exposure, number of discharges, pulmonary venous catheter operation time, effective ablation time for the left pulmonary vein, immediate success rate of intraoperative pulmonary venous isolation, postoperative maintenance rate of sinus rhythm, and recurrence rate of atrial fibrillation (AF). Recurrence was defined as the presence of AF and atrial tachycardia (AT) detected through a 12-lead ECG or long-term ECG monitoring three months after ablation, specifically encompassing instances where AF/AT lasted for more than 30 s. This assessment incorporated data obtained from various sources, including cardiac implantable electronic devices, among other pertinent considerations.

Safety endpoints: perioperative complications encompassing acute thromboembolic events (ischemic stroke, transient cerebral ischemic attack, peripheral artery embolism, and so on), pericardial effusion and cardiac tamponade necessitating drainage and surgical intervention, vascular puncture complications, esophageal injury, coronary and pulmonary vascular injury, phrenic nerve palsy, sinus node injury, and atrioventricular block.

### Statistical analysis

Measurement data that conform to the normal distribution are expressed as mean ± standard deviation, whereas those that do not conform to the normal distribution are expressed as median + interquartile range. The counting data are expressed as percentages. The Chi-squared test or Fisher’s exact test was used for the counting data, whereas two-independent sample *t*-test or ANOVA was used for the measurement data. The nonparametric test was used for data that do not conform to the normal distribution. The two-sided test was adopted, and *P* < 0.05 was considered statistically significant.

## Results

**Fig. 3:**
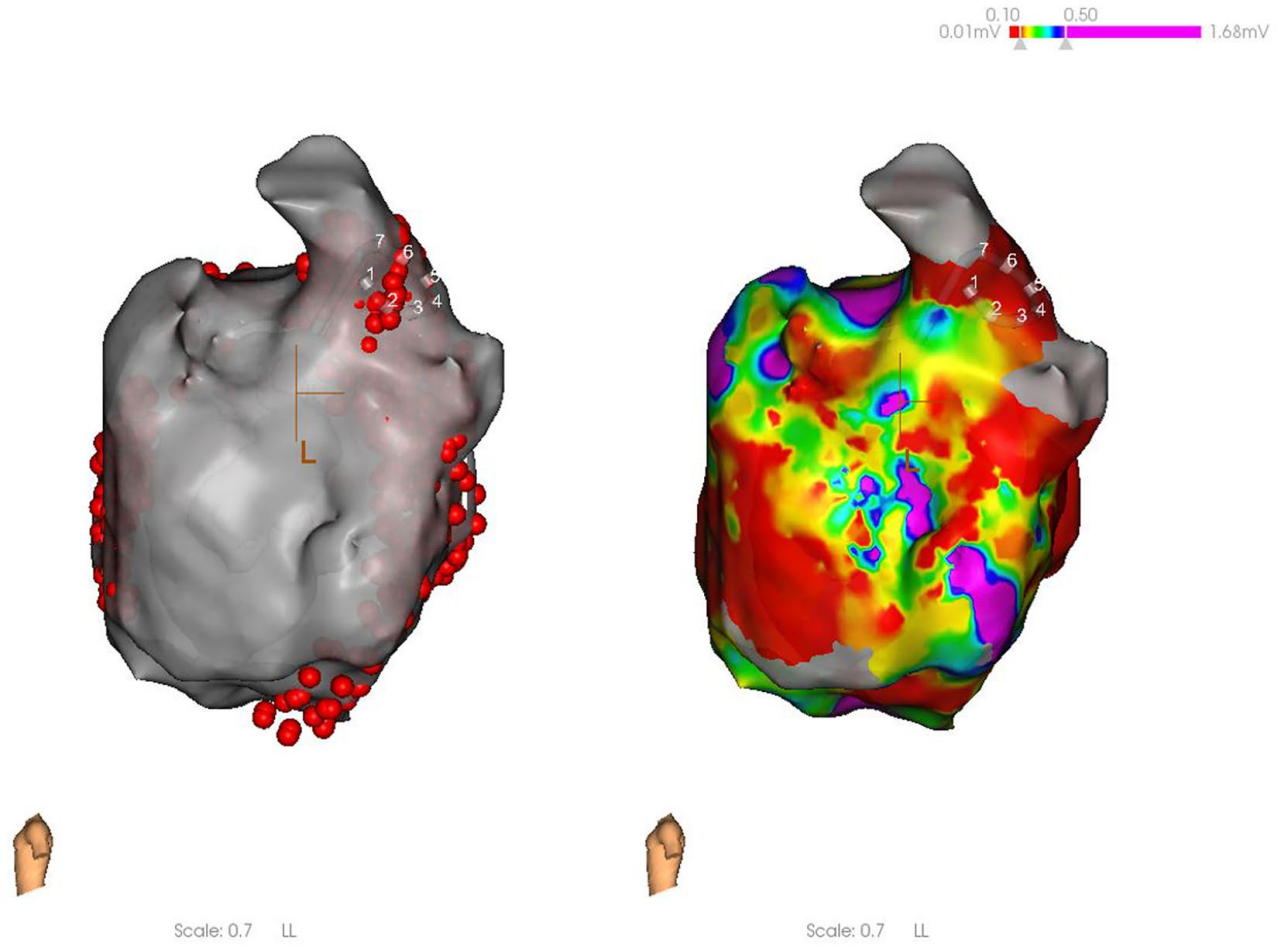
Anterior wall ablation (LL)

**Fig. 4:**
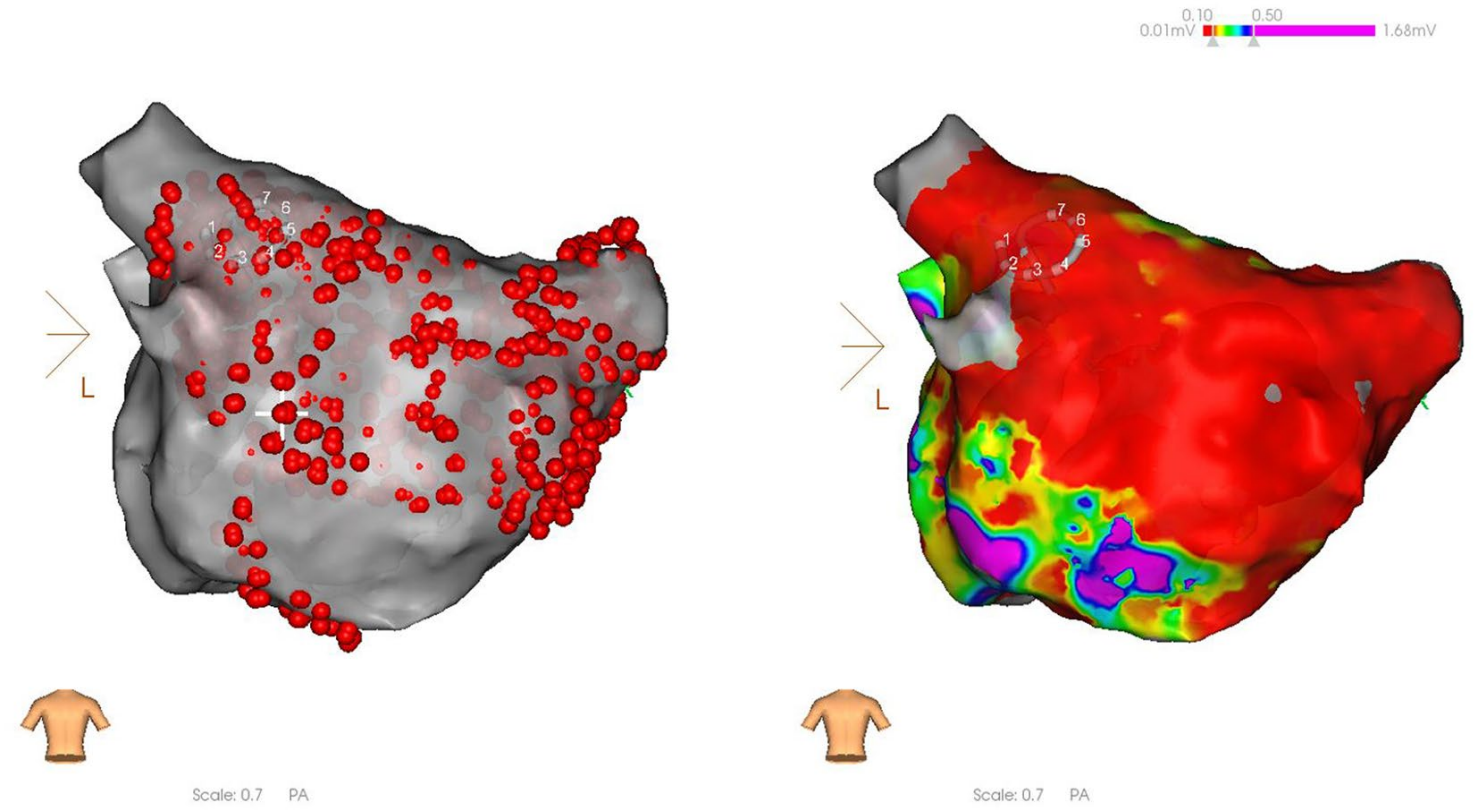
Posterior wall ablation

**Fig. 5:**
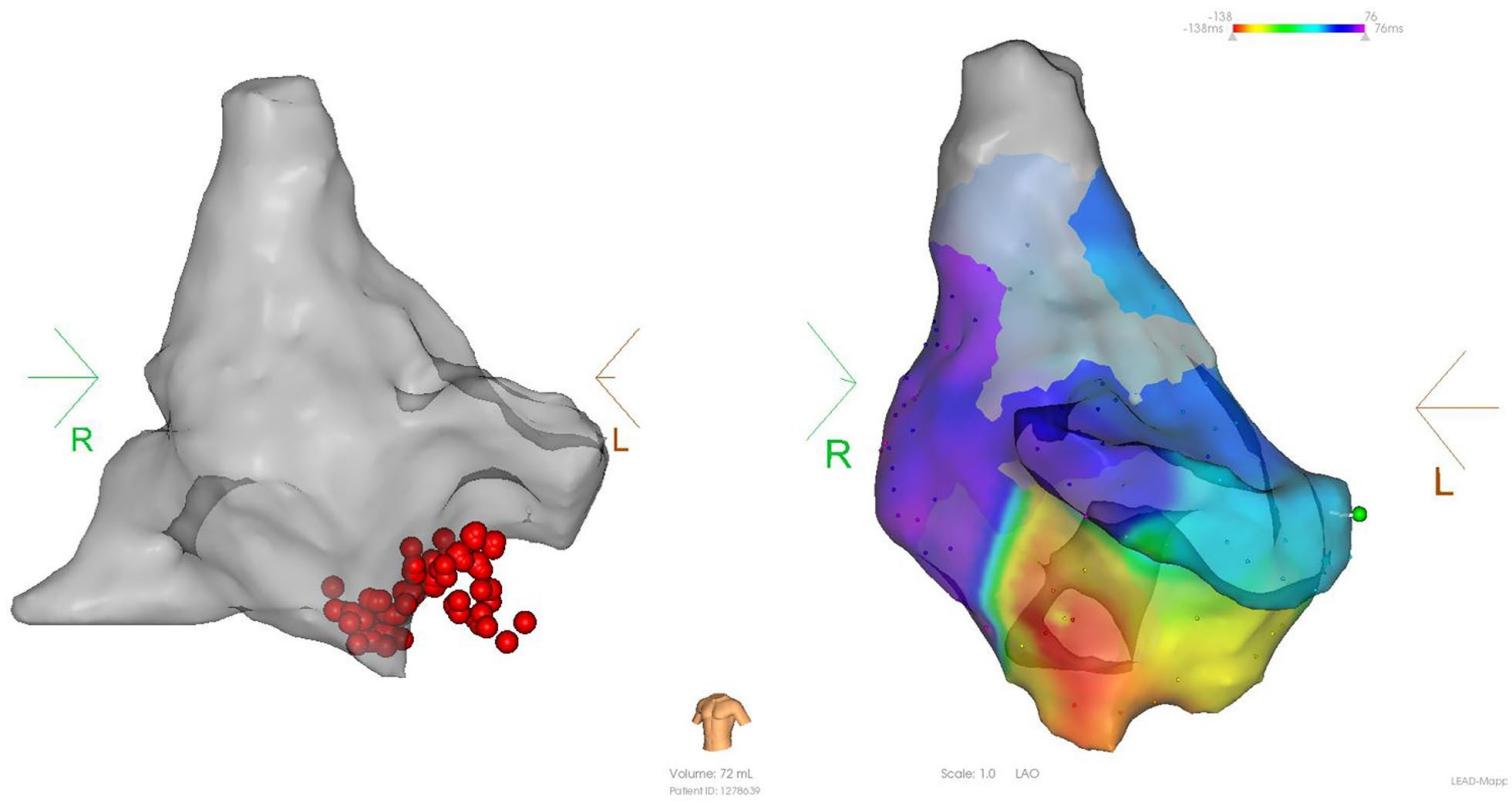
Cavotricuspid isthmus line block

**Fig. 6:**
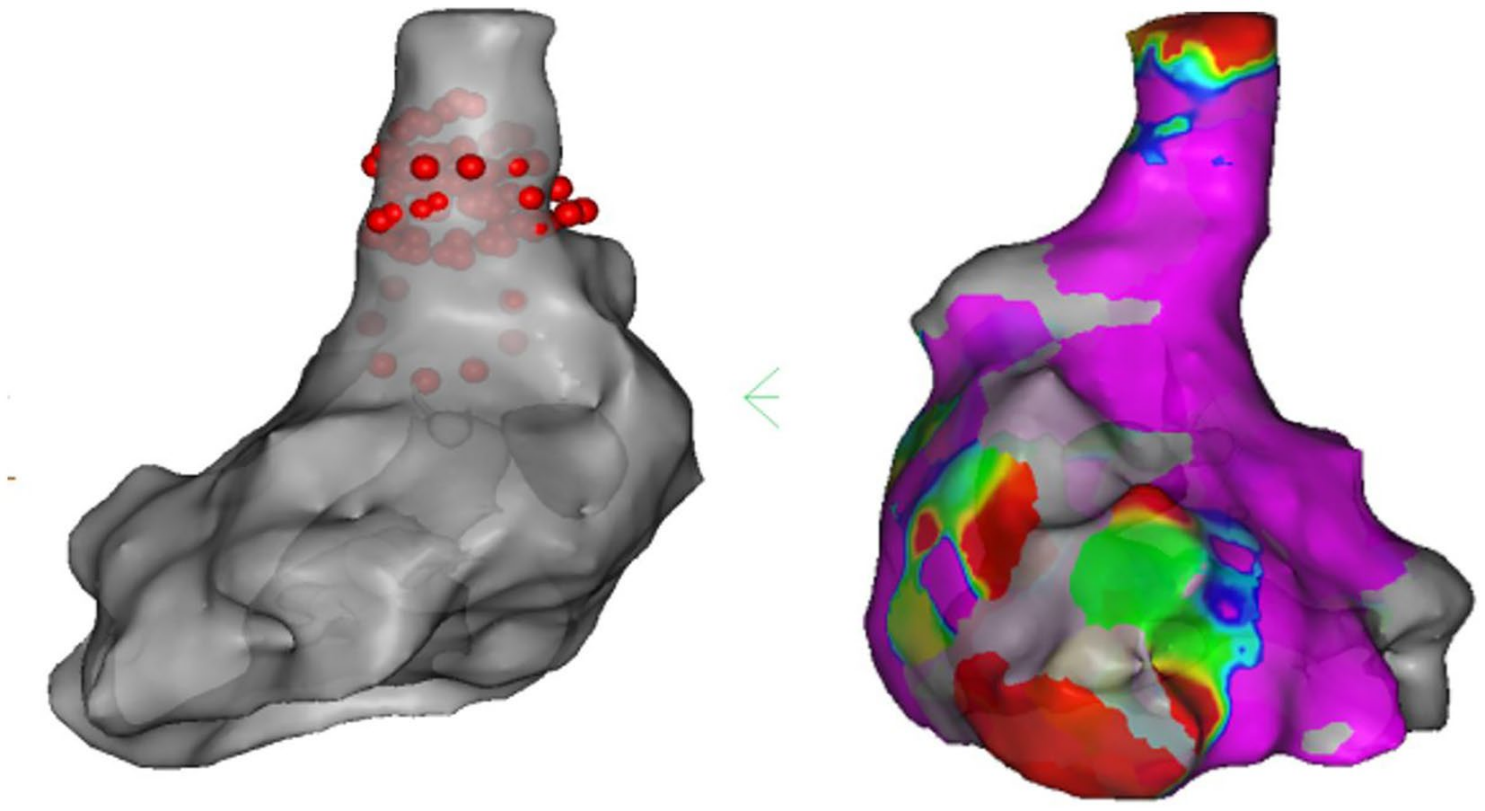
Superior vena cava isolation

**Fig. 7:**
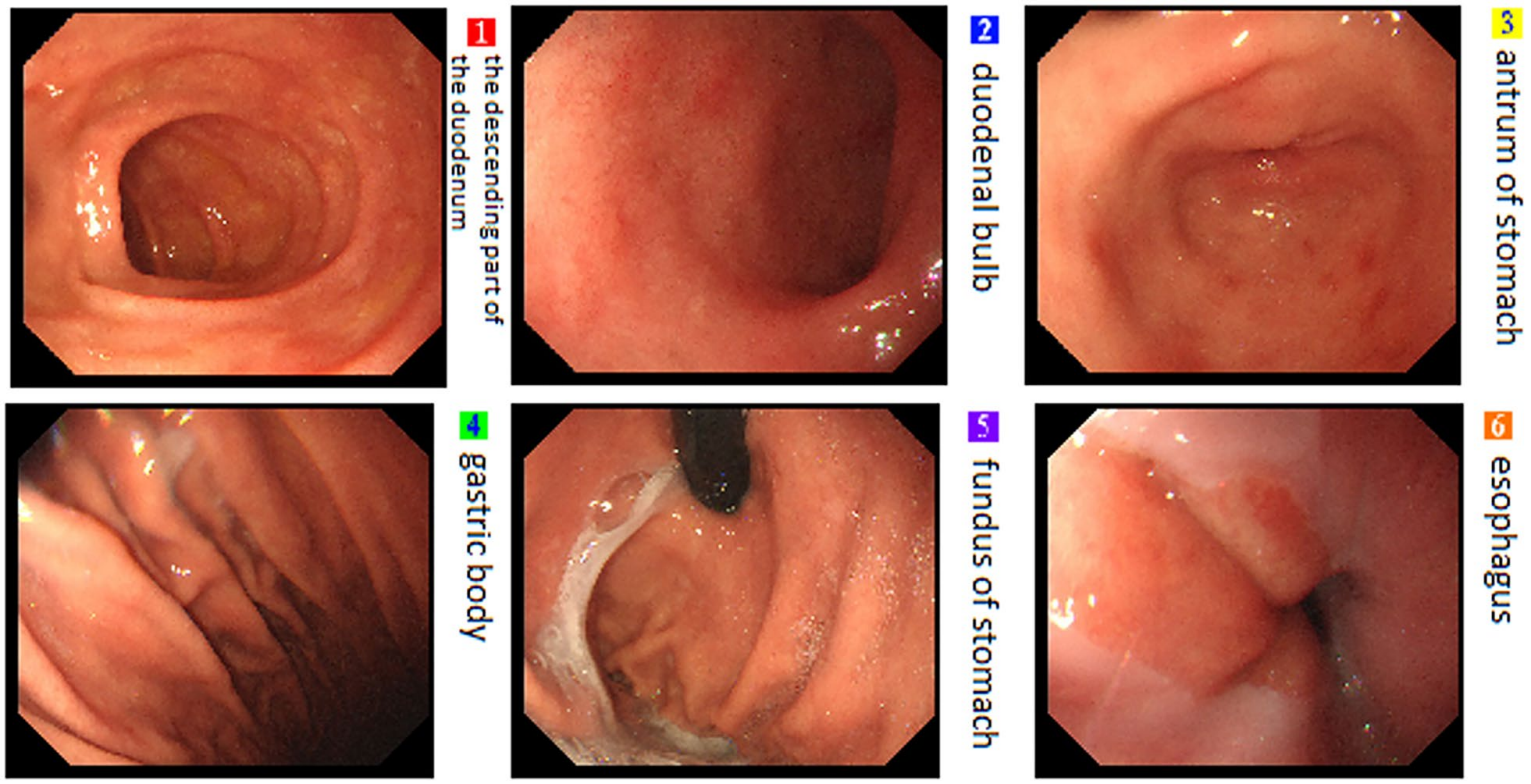
Postoperative gastroscopy

1. No significant differences were observed in the baseline data, including gender, age, weight, left atrial diameter, LVEF, CHS2DS2-VASc score, HAS-BLED score, AF type, history of hypertension, diabetes, and stroke, and the use of non-vitamin K antagonist oral anticoagulants (NOAC) and class I, II, and III antiarrhythmic drugs (*P* > 0.05, Table 1).
2. No significant differences were observed in the success rate of immediate PVI between the PFA group and the radiofrequency ablation group (*P* > 0.05). The effective ablation time of the left and right pulmonary veins were significantly shorter in the PFA group than in the radiofrequency ablation group (all *P* < 0.001). No significant differences were observed between the two groups in left and right pulmonary venous catheter operation time, retention time of catheter in left atrium, operation time, or radiation volume (*P* > 0.05, Table 2).
3. In the PFA group, the left pulmonary vein had a significantly higher number of discharges, catheter operation time, and effective ablation time than the right pulmonary vein (*P* < 0.05). In the radiofrequency ablation group, the number of left pulmonary vein discharges was significantly higher than that of the right pulmonary vein (*P* < 0.05). No significant differences were observed between the catheter operation time and effective ablation time of the left and right pulmonary veins in the radiofrequency ablation group (*P* > 0.05) (Table 3). In the PFA group, the number of discharges and effective ablation time of each pulmonary vein were significantly different (*P* < 0.001), with the left lower pulmonary vein, requiring significantly more effective ablation time (*P* < 0.001) and discharges (*P* < 0.001) during left pulmonary vein ablation, and the differences were statistically significant. As for right pulmonary vein ablation, there were statistically significant differences in the increase in effective ablation time (*P* = 0.041) and number of discharges (*P* = 0.027, Table 4).
4. When comparing sinus rhythm maintenance and AF recurrence rate at 1, 3, and 6 months of postoperative follow-up, no statistically significant differences were observed between the two groups for paroxysmal, persistent, and paroxysmal + persistent AF (*P* > 0.05, Table 5).
5. In the PFA group, 12 cases (33.3%) of anterior wall line, 14 cases (38.9%) of posterior wall isolation, 6 cases of cavotricuspid isthmus line block (16.7%), and 16 cases (44.4%) of superior vena cava isolation were successfully ablated (Figs. 3, 4, 5, and 6).
6. After PFA, gastroduodenoscopy was performed on 12 patients (33.3%) and neither esophageal nor fundus perforation or bleeding were observed (Fig. 7).
7. No complications were observed in either the PFA group or the radiofrequency ablation group, including acute thromboembolic events (ischemic stroke, transient cerebral ischemic attack, peripheral artery embolism, etc.), pericardial effusion and cardiac tamponade requiring drainage and surgical intervention, vascular puncture complications, esophageal injury, coronary and pulmonary artery vascular injury, phrenic nerve palsy, sinus node injury, atrioventricular block, and other related complications.
8. Two patients in the PFA group experienced unplanned recurrences of AT and subsequently underwent successful repeated cardiac radiofrequency ablation three months later. Intraoperative electrophysiological examination of the left atrium revealed that the majority of the venous potential in both pulmonary veins had been restored.

**Table 1:** Comparison of baseline data

Indicators	PFA group (n = 36)	Radiofrequency ablation group (n = 36)	F value	*P* value
Male [n (%)]	26 (72.2)	23 (63.9)	–	1.000
Age (year)	68.36 ± 15.07	64.64 ± 14.43	0.560	0.560
Weight (kg)	60.91 ± 9.08	67.55 ± 9.42	− 1.682	0.108
Left atrial inner diameter (mm)	34.91 ± 7.60	33.64 ± 9.51	0.347	0.732
Left ventricular ejection fraction (%)	59.27 ± 11.05	59.18 ± 8.96	0.210	0.983
CHS2DS2-VASc score (points)	2.82 ± 1.83	2.73 ± 1.95	0.113	0.912
HAS-BLED score (points)	2.42 ± 2.02	2.00 ± 1.61	0.584	0.566
Paroxysmal atrial fibrillation [n (%)]	21(58.3)	19 (52.8)	–	1.000
Hypertension [n (%)]	19 (52.8)	16 (44.4)	0.001	1.000
Diabetes [n (%)]	0 (0.0)	3(8.3)	–	0.476
Coronary heart disease [n (%)]	10 (27.8)	6 (16.7)	–	1.000
Previous stroke [n (%)]	10 (27.8)	7 (19.4)	–	1.000
NOAC [n (%)]	36 (100.0)	36 (100.0)	–	–
Class I antiarrhythmic drugs [n (%)]	0 (0.0)	2(5.6)	–	1.000
Class II antiarrhythmic drugs [n (%)]	23 (63.9)	24 (66.7)	–	1.000
Class III antiarrhythmic drugs [n (%)]	13 (36.1)	7 (19.4)	–	0.635

**Table 2:** Comparison of intraoperative parameters

Indicators	PFA group (*n* = 36)	Radiofrequency ablation group (*n* = 36)	*F* value	P value
PVI immediate success rate	36(100.0)	36(100.0)	**–**	1.000
Operation time (min)^a^	98 ± 24.45	108.64 ± 35.57	− 0.817	0.423
Retention time of catheter in left atrium^b^ (min)	55.13 ± 12.36	67.60 ± 26.18	− 1.429	0.168
Radiation volume (mgy)	20.45 ± 7.12	24.54 ± 5.92	− 1.465	0.158
Number of discharges^c^ (time)	141.51 ± 85.70	91.82 ± 15.63	19.514	< 0.001
Number of discharges in the left pulmonary veins (time)	89.41 ± 70.23	50.48 ± 12.77	18.763	< 0.001
Number of discharges in the right pulmonary veins (time)	52.10 ± 16.84	41.63 ± 5.92	19.902	< 0.001
Pulmonary venous catheter operation time^d^ (min)	37.35 ± 7.36	38.64 ± 8.19	− 3.900	0.701
Left pulmonary venous catheter operation time (min)	20.83 ± 5.22	20.18 ± 6.94	2.470	0.807
Right pulmonary venous catheter operation time (min)	16.52 ± 3.97	18.46 ± 3.34	− 1.242	0.229
Effective ablation time^e^ (ms)	49.76 ± 24.03	1,448,890.91 ± 397,502.59	− 12.089	< 0.001
Effective ablation time of the left lung (ms)	28.35 ± 20.02	769,200.00 ± 285,216.04	− 8.944	< 0.001
Effective ablation time of the right lung (ms)	21.41 ± 5.38	679,690.91 ± 1,771,785.11	− 13.122	< 0.001

The operation time refers to the length of time between the beginning of vascular puncture and the end of surgery

^b^Retention time of catheter in left atrium refers to the time between catheter insertion into the left atrium and catheter removal from the left atrium following successful ablation

^c^The number of discharges means the amount of pulse or ablation energy released each time the discharge pedal is pressed

^d^Pulmonary venous catheter operation time refers to the time required for pulmonary venous isolation, as computed by the system, which includes the time required for catheter movement and ablation

^e^The effective ablation time is the effective energy release time of the catheter, as calculated by the system

**Table 3:** Comparison of the left and right pulmonary vein ablation parameters for the radiofrequency and PFA groups

	Left pulmonary vein	Right pulmonary vein	*F* value	*P* value
Number of discharges in the radiofrequency group (time)	50.48 ± 12.77	41.63 ± 5.92	21.218	< 0.001
Number of discharges in the PFA group (time)	89.41 ± 70.23	52.10 ± 16.84	8.985	< 0.001
Pulmonary venous catheter operation time in the radiofrequency group (min)	20.18 ± 6.94	18.46 ± 3.34	0.741	0.467
Pulmonary venous catheter operation time in the PFA group (min)	20.83 ± 5.22	16.52 ± 3.97	2.180	0.041
Effective ablation time in the radiofrequency group (ms)	12.82 ± 4.75	11.33 ± 2.86	0.892	0.383
Effective ablation time in the PFA group (ms)	28.35 ± 20.02	21.41 ± 5.38	6.573	< 0.001

**Table 4:** Comparison of pulmonary vein branch parameters in the PFA group

Indicators	Left upper pulmonary vein	Left lower pulmonary vein	Right upper pulmonary vein	Right lower pulmonary vein	*F* value	*P* value
Number of discharges (time)	42.39 ± 25.26	61.91 ± 49.90	28.29 ± 10.10	22.22 ± 6.14	94.416	< 0.001
Pulmonary venous catheter operation time (min)	11.53 ± 2.33	9.30 ± 3.46	9.45 ± 2.98	7.07 ± 1.46	5.136	0.004
Effective ablation time (ms)	18.45 ± 8.84	24.38 ± 18.93	13.09 ± 3.77	10.08 ± 2.26	41.024	< 0.001

**Table 5:** Comparison of postoperative sinus rhythm maintenance

Indicators	PFA group (*n* = 36)	Radiofrequency ablation group (*n* = 36)	*P* value
1 month after operation [n (%)]	33 (91.7)	30 (83.3)	1.000
3 months after operation [n (%)]	33 (91.7)	33 (91.7)	1.000
6 months after operation [n (%)]	33 (91.7)	30 (83.3)	1.000
1 month after paroxysmal atrial fibrillation surgery [n (%)]	18 (85.7)	19 (100.0)	1.000
3 months after paroxysmal atrial fibrillation surgery [n (%)]	18 (85.7)	19 (100.0)	1.000
6 months after paroxysmal atrial fibrillation surgery [n (%)]	18 (85.7)	16 (84.2)	1.000
1 month after persistent atrial fibrillation [n (%)]	15 (100.0)	10 (58.8)	0.444
3 months after persistent atrial fibrillation [n (%)]	15 (100.0)	14 (82.3)	1.000
6 months after persistent atrial fibrillation [n (%)]	15 (100.0)	14 (82.3)	1.000

## Discussion

This study represents a real-world case analysis, aligning closely with clinical practice in both study design and follow-up procedures. It lays a robust foundation and accrues valuable insights for the future application of PFA in the treatment of AF. Furthermore, the implementation of propensity score matching in the selection of radiofrequency ablation case controls in this study, significantly mitigated the impact of confounding variables, thereby enhancing the reliability of the results. Through the observation of patients with AF undergoing PFA, initial insights into the efficacy and safety of this treatment modality have been obtained. Our analysis primarily focused on intraoperative parameters including operation time, catheter retention time in the left atrium, radiation volume, number of discharges, pulmonary venous catheter operation time, effective ablation time of the pulmonary veins, immediate success rate of intraoperative PVI, postoperative sinus rhythm maintenance rate, AF recurrence rate, and so on. However, objectively speaking, the rate of postoperative sinus rhythm maintenance and the rate of AF recurrence are crucial clinical endpoints. In comparison to the radiofrequency ablation group, the postoperative sinus rhythm maintenance rate and AF recurrence rate were found to be similar. However, statistically, PFA appears to have a lower recurrence rate, with sinus rhythm maintained in up to 91.7% of cases during the 3- and 6-month postoperative follow-up periods. This rate is slightly higher than the 78.5% observed in the overall paroxysmal AF cohort and the 85% in the optimized biphasic energy PFA waveform cohort, as estimated by the 1-year Kaplan–Meier analysis in the PEFCAT and PEFCAT II studies [12]. These figures are, however, comparable to the 6% recurrence rate documented in patients diagnosed with AF (*n* = 191) in the real-world study conducted by Schmidt et al. [13] While this study may lack extensive large-scale follow-up data, it is undeniable that patients have reported highly positive feedback regarding their clinical symptoms and surgical experiences during the follow-up period. Furthermore, it is worth noting that most recurrence events in the PFA group were AT, a finding consistent with the results from the study conducted by Tohoku et al. In their research, AT was identified as the predominant type of arrhythmia among the 25 patients who underwent a repeat operation [14]. Three months after PFA, a patient who experienced recurrent AT underwent successful treatment with radiofrequency ablation. Activation mapping revealed that the AT originated in the superior vena cava. Prior to the mapping, the patient had superior vena cava pulse isolation, but during the mapping, the upper cavity potential was fully restored. Furthermore, in additional matrix mapping, the conduction in both left and right pulmonary veins was substantially restored. Hence, there are lingering concerns about the long-term durability of pulsed pulmonary venous and superior cavity isolation. The results from the 1-year follow-up of the PEFCAT and PEFCAT II studies disclosed notable differences in the quantitative time analysis of pulmonary venous isolation [15]. In terms of safety, neither group of patients encountered acute thromboembolic events (such as ischemic stroke, transient cerebral ischemic attack, peripheral artery embolism, and so on). In some patients who underwent PFA, the presence of microbubbles was monitored using intraoperative intraluminal ultrasound. Additionally, no instances of pericardial effusion and cardiac tamponade necessitating drainage and surgery, vascular puncture complications, or coronary and pulmonary vascular injuries were reported. These findings underscore a high level of safety associated with the procedure. The results are in line with those reported in current clinical studies [12, 13, 16, 17]. However, there were two patients who experienced transient intersection escape rhythm with third-degree atrioventricular block (atrial pacing cannot be transmitted downwards) during the ablation of the posterior wall of the left pulmonary vein. Following PFA, ventricular electrodes were subsequently positioned to safeguard ventricular pacing in patients. A post hoc analysis indicated a potential connection between the abundant ganglion in the posterior wall of the left pulmonary vein and vagal reflex, with an incidence rate of 27.3%. Both animal experiments and clinical studies have documented the ablation of the superior vena cava, cavotricuspid isthmus line, mitral isthmus line, and left atrial posterior wall line. These studies have verified the feasibility of additional ablation procedures beyond PVI [18–20]. In the PFA group, successful exploration was achieved in 12 cases (33.3%) of anterior wall line, 14 cases (38.9%) of posterior wall isolation, 6 cases (16.7%) of cavotricuspid isthmus line block, and 16 cases (44.4%) of superior vena cava isolation. Notably, one patient with cavotricuspid isthmus-associated atrial flutter was effectively ablated and terminated through cavotricuspid isthmus line ablation, and another patient with superior vena cava-originating atrial tachycardia was successfully ablated via superior vena cava isolation. In contrast to the local micro-pulsed ablation catheter, the Jinjiang circular catheter utilizes a pulse-released electrode to achieve the objective of local selective ablation. Despite the addition of further ablation to the PVI procedure, no additional complications were reported, including esophageal injury (noted in 33.3% of patients, including those who underwent posterior wall ablation and required postoperative gastroduodenoscopy), phrenic nerve seizures during ablation, phrenic nerve palsy, sinus node injury, or atrioventricular block during the follow-up period. Overall, PFA demonstrated high sinus rhythm maintenance, a low recurrence rate, and high safety in patients with paroxysmal or persistent AF. Apart from animal experiments, the feasibility of additional PVI ablation has been consistently demonstrated in patients undergoing AF ablation, yielding positive outcomes. However, in PFA, akin to radiofrequency ablation, the selection of ablation energy may influence the long-term durability of isolation. Presently, PFA energy selection relies primarily on evidence from animal experiments, lacking substantial patient data on a large scale, necessitating further investigation and research.

Comparing quantitative intraoperative parameters in experimental studies can offer valuable insights into the use of PFA catheters. Variations in operation time among different centers could arise from diverse operating practices. However, most studies have reached a consensus that PFA typically demands a shorter surgical operation time when compared to other ablation methods. However, the results of this study reveal minimal differences in operation time between the PFA and radiofrequency ablation groups. Additionally, there were no statistically significant distinctions in catheter retention time in the left atrium and operation time for the pulmonary venous catheter. This observation could be attributed to the matrix mapping being executed both before and after ablation in the PFA group, a procedure that was seldom performed in the radiofrequency ablation group. An intraluminal ultrasound catheter was used in some patients in the radiofrequency ablation group, allowing for the pre-ablation reconstruction of the left atrial model for three-dimensional anatomical mapping. Although intraluminal ultrasound was used in some patients in the PFA group, it was used in conjunction with the left atrial appendage occlusion after ablation, without the need for model reconstruction. This approach indirectly highlights the remarkable efficiency of the pulsed annular electrode for left atrial modeling and matrix mapping in PFA procedures. PFA also demonstrated a perfect immediate success rate in PVI and required significantly shorter effective ablation time. Leveraging the insights derived from experiences with radiofrequency ablation, it is recognized that a shorter adherent ablation time can mitigate the risks associated with steam bursts and heart rupture. Nonetheless, in the surgical process, the PFA group required more discharges than the radiofrequency ablation group. This phenomenon could be linked to the early recovery of isolation conduction observed in the PFA group. During the post-ablation observation period, it was noted that the potential capacity of the anterior wall of the left pulmonary vein and the top of the right pulmonary vein recovered in most patients. Consequently, repeated ablation was necessary until complete isolation was confirmed. This iterative process led to an increase in both the operation time and the number of discharges. Intraoperative statistics revealed that the operation time of the left pulmonary vein catheter in the PFA group was longer than that of the right pulmonary vein, and it was the same in the radiofrequency ablation group, indicating that the operation time of the pulsed ablation catheter was still limited by the influence of the puncture point and axial direction. It was observed that isolating the left pulmonary vein was more difficult than isolating the right pulmonary vein. Based on a detailed analysis of the PVI process in PFA, it was found that the left superior pulmonary vein required the most time within the left pulmonary vein, whereas the right superior pulmonary vein posed the greatest challenge within the right pulmonary vein. These specific areas are inherently challenging to access during the standard operational procedure. Furthermore, the absence of a tissue adhesion reminder and pressure monitoring in the pulsed ablation catheter can lead to a rise in ineffective adhesion and discharge. This factor may also account for the relatively straightforward recovery of potential conduction in these specific regions. In summary, PFA offers rapid and immediate PVI, thanks to the multi-electrode and annular design of the catheter, which enhances surgical efficiency. However, there is potential for improvement in intraoperative adhesion feedback, pressure monitoring, ablation AI value monitoring, and other aspects. These enhancements are crucial to minimize interference and axial impact on the atrial septal puncture position, as well as to mitigate ineffective discharges and excessive tissue damage. The transition from a radiofrequency ablation catheter to a pulsed catheter was seamless and swift, with surgeons quickly adapting to its usage and mastering the operational skills. This process required no more time than that for radiofrequency ablation, underscoring the shorter learning curve associated with PFA. This observation aligns with the comparisons drawn from similar experiments, confirming the efficiency of PFA in terms of learning and adaptation [13, 21, 22].

This study is subject to several limitations. The sample size was limited, and individual differences among cases may have introduced a lack of representativeness. Despite employing propensity score matching in the selection of patients for the control group, which substantially minimized potential biases between the two groups, there remain disparities compared to randomized controlled studies.

## Conclusion

In summary, over the 6-month follow-up period, PFA for both paroxysmal and persistent atrial fibrillation exhibits outcomes comparable to radiofrequency ablation in terms of recurrence rate, safety, surgical efficiency, and a shorter learning curve.

## Data Availability

The datasets used or analyzed during the current study are available from the corresponding author on reasonable request.
